# Study protocol for a randomized controlled trial investigating the effect of online interpretation bias intervention on stress reactivity in the children of parents with depression: the CoCo study

**DOI:** 10.1186/s12888-023-04803-y

**Published:** 2023-05-30

**Authors:** Tonya Frommelt, Leonie Bäumler, Nicolas Rohleder, Gerd Schulte-Körne, Belinda Platt

**Affiliations:** 1 Department of Child and Adolescent Psychiatry, Psychosomatics and Psychotherapy, LMU University Hospital Munich, Nussbaumstr. 5, 80336 Munich, Germany; 2 Department of Psychology, Chair of Health Psychology, Friedrich-Alexander-University Erlangen-Nürnberg, Nägelsbachstr. 49a, 91052 Erlangen, Germany

**Keywords:** Paediatric, Adolescent, Psychiatry, Interpretation bias training intervention, Cognitive behavioural therapy, Parental depression, RCT, Offspring, Preventive

## Abstract

**Background:**

Current preventive interventions for the children of parents with depression demonstrate modest effects on depression incidence. This may be because existing interventions tend to comprise general psychotherapeutic tools, rather than targeting the specific mechanisms underlying familial transmission. Improved theoretical models of familial transmission could enhance the development of targeted interventions. Although existing models assume that cognitive and biological vulnerability factors influence one another, the precise mechanisms are unknown. This project is the first to experimentally test whether negative interpretation bias has an impact on cortisol response in children of parents with depression. This study protocol reports a randomised controlled trial of an interpretation bias intervention which aims to shift participants’ interpretation bias in a more positive direction and thereby alter their stress response.

**Methods:**

Children aged 10–14 years who have i) one parent with a current or previous depression diagnosis, with at least one episode occurring during the child’s lifetime and ii) do not have a current or previous psychiatric diagnosis themselves, will be assigned to one of two conditions: an interpretation bias intervention (*n* = 50) or a structurally similar placebo intervention (*n* = 50). The interpretation bias intervention consists of a short lab-based cognitive reappraisal of interpretations training, a four-week app-based Cognitive Bias Modification of Interpretations intervention and interpretation bias specific if–then plans. Interpretation bias will be assessed before and after the intervention using the Scrambled Sentences Task. The effect of the intervention on participants’ stress response will be assessed by salivary cortisol collected at five different time points: from immediately before until 45 min after administering the Trier Social Stressor Test for Children. Stress reactivity will be measured via baseline to peak cortisol and stress recovery will be measured via the 45 min cortisol marker. We hypothesise that children who participate in the interpretation bias intervention will display a positive shift in interpretation bias and this, in turn, will alter their stress response. Children who receive the placebo intervention are expected to show a smaller positive shift in interpretation bias and stress reactivity.

**Discussion:**

The findings of the present study will contribute to models of familial depression transmission as well as informing preventive interventions. If training a more positive interpretation bias subsequently alters participants’ stress response, then incorporating such tools may increase the efficacy of existing preventive interventions.

**Trial registration:**

Deutsches Register Klinischer Studien DRKS00028842. Registered August 19, 2022.

## Background

Depression is one of the most common mental disorders with lifetime prevalence rates of 13- 16% [1, 2]. Adolescence is considered a highly sensitive period for developing a depressive episode for the first time [3–5]. In addition to biological changes, psychological and social factors have a notable influence on adolescents [6, 7]. Developing depression during this critical period can have a severe impact on individuals’ life trajectory. Impaired functioning during a depressive episode directly affects school completion, career progression, and integration into the social environment [3, 8]. A depressive episode in children and adolescents is also associated with an increased risk of suicide and chronification of the disease [9]. More effective preventive interventions which target groups at elevated risk are needed if the burden of depression is to be reduced [10].

One of the biggest risk factors for depression is having a parent who has experienced depression: children of depressed versus non-depressed parents are more likely to suffer from depression 20 years later (65% vs. 27%; RR = 3.37) [1]. This risk is thought to be conveyed both via biological pathways (e,g., genetic factors and dysfunctional neuroregulation during pregnancy) as well as environmental pathways (e.g., parenting and adverse life experiences) which together increase depression vulnerability amongst offspring [11]. Cross-sectional studies have identified several vulnerabilities in the children of parents with depression (hereafter referred to as “high-risk”; HR) compared with children of parents with no mental health history (“low risk”; LR) [11]. For example, HR versus LR children show difficulties in emotion regulation [12–14], maladaptive cognitive styles [15, 16], dysfunctional social skills [13, 17] and psychobiological alterations in the hypothalamic–pituitary–adrenal (HPA) axis [15, 16, 18]. In line with diathesis-stress models of depression, these vulnerability factors are thought to remain latent unless triggered by stress. Although Goodman and Gotlib state that these affective, cognitive, behavioural and psychobiological vulnerability factors “…will almost certainly interact and affect one another” ([11] p. 460, para. 5), few studies have addressed causal relationships between these vulnerability factors. Understanding the relationship between these vulnerability factors is not only of theoretical importance but may help to develop more targeted interventions. Existing preventive interventions tend to use general psychotherapeutic methods (e.g. psychoeducation, behavioural activation) and show relatively modest effects on the incidence of depression [19–21].

The current study addresses the interplay between two of the most established vulnerability factors for depression amongst HR youth: cognitive vulnerability and cortisol stress reactivity. Cognitive vulnerability is defined as the tendency to adopt a maladaptive cognitive style following a stressful event. A number of cognitive styles have been identified as maladaptive for mental health [22], including negative attributional style (attributing negative events to personal versus external factors) [23], negative schemas (negative thoughts about the self, world and future) [24–26] and negative response styles (focusing on the cause of a negative event rather than solutions) [27, 28]. The focus of this study is on the role of negative cognitive biases: the tendency to prioritise negative (over neutral or positive) material [29]. Cognitive biases can be readily measured using behavioural tasks which, by measuring cognition under mental load, minimise response biases [29]. These relatively automatic negative biases are thought to maintain a number of maladaptive cognitive styles e.g., negative schemas. Although negative cognitive biases can occur at the level of attention (AB), interpretation (IB) and memory (MB) [29, 30], the focus in this study is on negative IB [31] since it is more reliably observed than AB [32] or MB [33] in relation to depression.

A negative IB can be demonstrated in depressed adults [29, 34–36] and youth [37] and prospectively predicts depression [38, 39]. Behavioural studies have found that HR children more often interpreted emotionally-ambiguous words [40] and sentences [41] in a negative manner. Importantly, this is the case even once children’s own symptoms of depression have been controlled for, suggesting that negative cognitive styles are not simply the by-product of inherited depression [42].

According to the perseverative cognition hypothesis, chronic patterns of negative thinking have a direct impact on the stress response via the HPA axis, a neuro-endocrine system which plays a key role in physiological stress [43, 44]. The HPA axis involves the hypothalamus, the pituitary gland and the adrenal glands. It responds to stress and controls many bodily processes including digestion, the immune system and emotions. The HPA response has three temporally distinct phases: 1) basal activity – baseline activity displaying a circadian rhythm in the absence of any stimulation, 2) reactivity phase – increase from basal levels in the 30 min following a stressor, 3) recovery phase – reduction of levels to baseline phase [45]. Cortisol reactivity and recovery is typically measured in the laboratory by exposing participants to a standardized social stressor e.g., the Trier Social Stress Test (TSST) [46] and measuring changes in salivary cortisol before, during and after the task. An early meta-analysis found depressed adults to show elevated cortisol levels in the recovery (but not reactivity) phase of a “very large” effect size [47]. However, these findings should be interpreted cautiously since the meta-analysis included just seven studies all of which had modest samples sizes (*n* = 7 – 23). Subsequent meta-analyses which suggest more modest effect sizes are also limited by small sample sizes and the inclusion of studies of depressed patients in remission [48, 49]. Some of the heterogeneity of these findings may also be due to limitations of the most commonly used means of analysing cortisol reactivity: Area Under the Curve (AUC). Since it takes the complete cortisol response into account, AUC cannot distinguish between cortisol reactivity and recovery [50]. A more direct operationalization of reactivity involves calculating delta values (change from baseline to peak cortisol activity) [48]. Other meta-analyses suggest cortisol reactivity in depressed adults is modified by sex [49] and basal cortisol [48]. Burke et al. further found a moderating role of time of day [47].

Sample sizes in studies of cortisol stress response in children and adolescents are generally higher. Some studies have found significantly higher cortisol levels in response to a social stressor in depressed versus non-depressed children [51, 52]. Other studies suggest that the direction of effects depends on the pubertal developmental status of the children [52–54], experiences of childhood maltreatment [55], the chronicity of depression [56, 57] and whether patients are medicated [58]. Cortisol response to stress may also be a marker of depression risk in adolescents. Altered cortisol stress reactivity predicts the later occurrence of depression in adolescents [53]. Furthermore, altered cortisol reactivity has been demonstrated in the healthy children of depressed parents [18, 59]. In the majority of studies, higher cortisol reactivity values were found in connection with a social stressor for children of depressed parents compared to children of non-depressed parents. Whilst two studies have found increased cortisol stress reactivity in pre-school [60] and school- aged [61] HR children, others have not [62, 63]. However, the stressors in these studies were relatively mild (e.g., a brief puff of air to participants’ throats) [62]. None of these studies used the TSST [46] (or TSST-C [44]), despite it being the gold-standard paradigm.

Although the perseverative cognition hypothesis asserts that cortisol response to stress is directly influenced by cognitions, relatively little research has experimentally tested this hypothesis. Experimentally reducing negative response styles was associated with reduced cortisol reactivity in an unselected adult sample [64]. One means by which cognitive biases can be experimentally manipulated is via Cognitive Bias Modification of Interpretations (CBM-I) paradigms [33]. In CBM-I studies, participants in the intervention condition show more positive interpretations compared to placebo or negative control conditions post intervention [65–67] and reduced negative affect [68]. A decrease in depression-typical cognitive patterns through CBM-I has also been observed in depressed adolescents and young adults [37]. Further studies found that CBM-I led to a decrease in psychophysical indicators of stress reactivity such as electrodermal activity [69] and heart rate [66, 69]. Reducing negative IB via Cognitive Bias Modification of Interpretations (CBM-I) training [70] has been associated with reduced heartrate variability and electrodermal activity in clinically depressed adults [71]. Studies of the association between cognitions and cortisol stress reactivity in children and adolescents are limited to cross-sectional and longitudinal designs. In two non-clinical samples, adolescents with prolonged cortisol recovery and increased cognitive vulnerability [72] or more stressful life events [73] showed higher depressive symptoms. Moreover, in depressed adolescents, pronounced rumination was associated with delayed cortisol recovery [74]. In a novel study of 561 unselected adolescents prolonged cortisol recovery after confrontation with an external stressor predicted the onset of a depressive episode three years later [75]. Just one study has investigated the association between cognition and cortisol stress response in children of parents with depression. Children of depressed (but not non-depressed) parents with low self-esteem, AB or MB showed increased cortisol reactivity two years later [15].

In the following we describe the study protocol (Version 1; February 2023) of the CoCo study, a randomised controlled trial (RCT) examining the connection between cognitive and psychobiological vulnerability in the familial transmission of depression. The research question being addressed is whether reducing cognitive vulnerability alters psychobiological reactions to stress. The following two hypotheses are to be tested in the RCT: i) Cognitive vulnerability, as indicated by a negative IB, can be made more benign through our IB intervention and ii) negative IB will affect psychobiological vulnerability such that training more benign IB will result in altered cortisol reactivity and recovery. We also assume that at baseline, HR will show altered IB and cortisol reactivity compared to LR children, however this hypothesis is not part of the main RCT. Children who receive the placebo intervention are not expected to show a positive shift in IB or show altered cortisol reactivity or recovery from pre to post IB intervention. To the best of the authors knowledge, this is the first study to investigate whether cognitive vulnerability may play a causal role in psychobiological reactions to stress in HR children.

## Methods/Design

This study protocol is reported in accordance with the SPIRIT 2013 Statement (Standard Protocol Items: Recommendations for Interventional Trials) [76]. The study has received ethical approval from the Ludwig-Maximilians-University (LMU) Medical Division Ethics Committee, Munich, Germany (Study ID: 19–691).

### Design

**Fig. 1:**
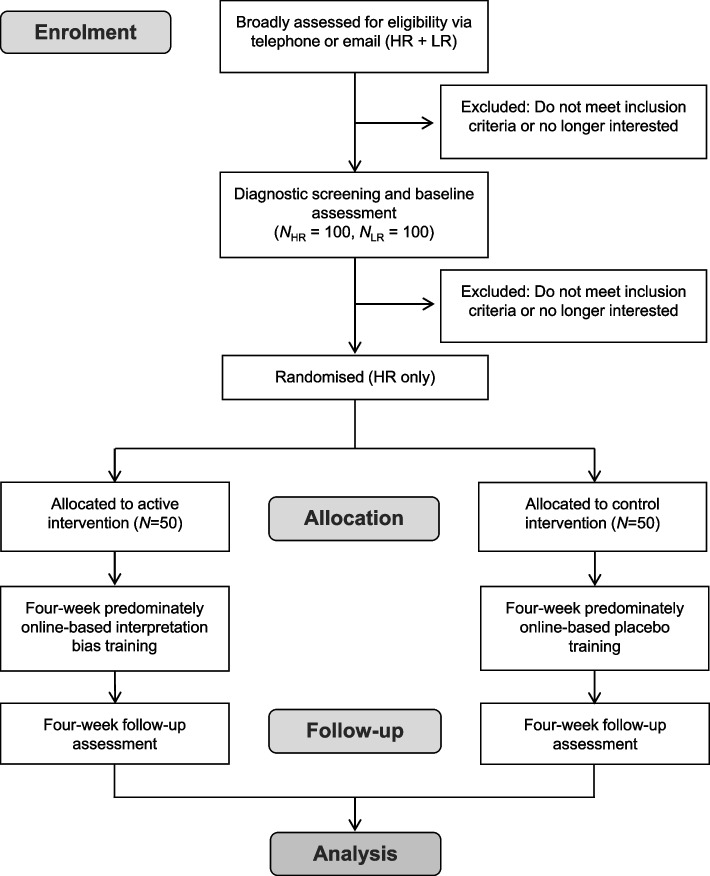
Overview of the Study Design. Note*:* HR = high risk children of depressed parents; LR = low risk children of non-depressed parents. Both HR and LR individuals are included in an initial baseline assessment session to determine differences in these samples in outcome measures. Only HR individuals are included in the RCT

Figure 1 depicts the study flow. In session one of this study (baseline assessment) both HR and LR individuals are included to determine differences in these samples in IB and cortisol reactivity and recovery. In session two of this study (RCT), only HR individuals are included. This study protocol manuscript focuses on the RCT component, which involves an IB intervention for 100 HR individuals. In a parallel groups design, the study will compare a four-week predominately online-based IB intervention (*N* = 50) with a temporally and structurally similar placebo intervention (*N* = 50).

Following an initial assessment session (T1), HR individuals will be randomised to one of the two intervention groups (IB intervention versus placebo intervention). Randomisation will take place after baseline assessment of measures and immediately before the commencement of the IB intervention. The experimenter (TF or other) will call the person in possession of a computer-generated random list of group memberships (BP) to obtain participants’ allocation status. The experimenter will not have access to the list.

Both intervention groups with HR individuals will take part in a baseline assessment session: (T1) immediately before the intervention and an outcome assessment session (T2) four weeks after baseline. LR individuals, who do not receive the intervention, will only take part in the baseline assessment session (T1). While the outcome assessor will be aware of the intervention condition which HR individuals are assigned to, participants themselves will not.

The laboratory testing sessions will take place in the research department of the Child and Adolescent Psychiatry of the LMU University Hospital in Munich. Data will be collected both at the University Hospital and from participant’s smartphones via the app-based component of the intervention created for this study.

### Participants

Participants should have i) at least one parent who meets the diagnostic criteria for major depression (according to the Diagnostic and Statistical Manual of Mental Disorders; DSM-5) for a current depressive episode or report having had a depressive episode in the participating child's lifetime that fulfils the diagnostic criteria (HR) or no parental history of depression (LR) and ii) be between 10–14 years of age. Adequate German-language skills are a further requirement due to the verbal nature of various study components.

Participants will be excluded from the study if i) either parent has a history or current symptoms of bipolar disorder, psychosis or substance abuse, or has severe symptoms of another disorder that could interfere with study outcomes, ii) the participating child meets diagnostic criteria for a current (or past) episode of a psychiatric disorder, iii) and the child is taking medication that could affect cortisol levels. Children who are in crisis or have severe symptoms of another disorder which could interfere with their ability to take part in this study may also be excluded (particularly in the case of HR children as this may interfere with the extensive intervention component of the RCT). Both parents (if applicable) and the participating child must provide written informed consent.

### Recruitment

Therapists in Munich and the surrounding area will be asked to inform suitable adult patients about the study. Advertisements will be placed in various locations throughout Munich, including in public spaces and general practises. Letters containing study flyers will be sent out to families whose address details are registered in the Munich district administration office. Persons who have participated in previous studies of the research group and have expressed willingness to participate in future studies will be contacted. HR participants who partake in all study sessions will receive €100 (€25 at T0, €25 at T1 [LR and HR], and €50 at T2 [HR]).

### Procedure

**Fig. 2:**
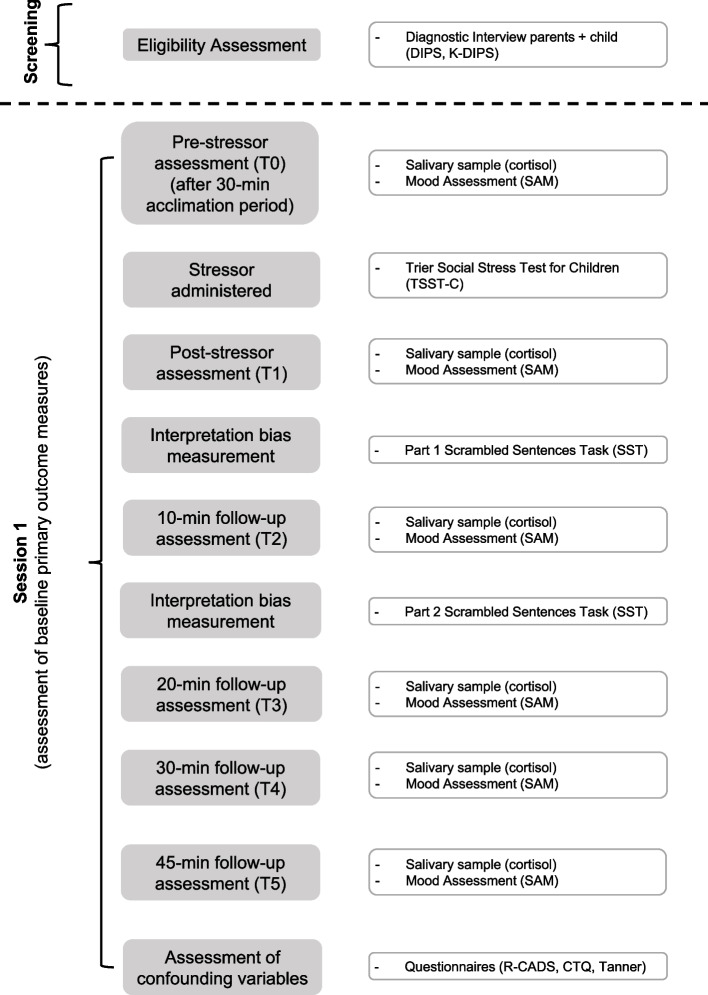
Overview of the Study Procedure: Participant Screening and Session 1. Note*:* Session 1 depicts the baseline assessment of primary outcome measures. Both HR and LR participants are included in session 1

**Fig. 3:**
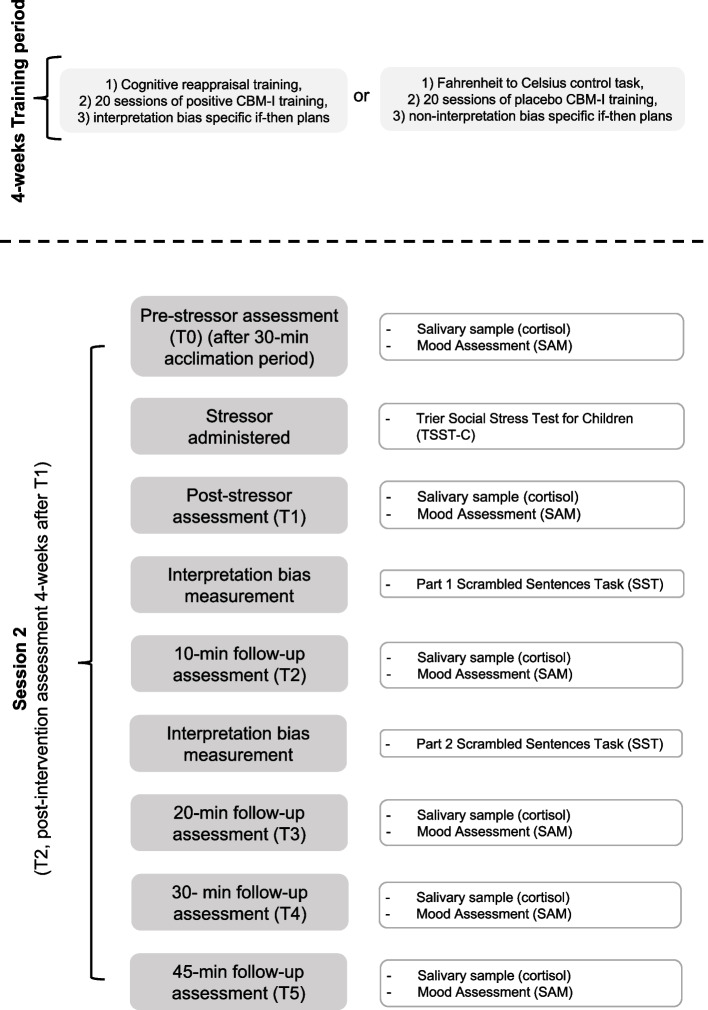
Overview of the Study Procedure: IB Intervention and Session 2. Note*:* Session 2 depicts the post- intervention assessment of outcome measures. Only HR participants will take part in the IB intervention and thus only HR participants are included in session 2

See Figs. 2 and 3 for an overview of the study procedure. Interested children and parents will initially be broadly screened on whether they fit the basic inclusion criteria by telephone or email. A diagnostic screening session (T0) will then be scheduled for eligible participants either in the laboratory or via video call. The non-attending parent (if applicable) will be interviewed separately via video call. The screening will be conducted and scored by a person trained and/ or experienced in conducting psychiatric evaluations. The child and both parents (if eligible) should provide written informed consent prior to the commencement of the screening session. At T0 the child and attending parent (affected parent in the case of HR individuals) will receive an overview of the study, including details on group randomisation in the case of HR individuals. After T0, a final decision will be made on the child's eligibility.

In the second lab session (T1), which will be scheduled no more than 12 weeks after screening (T0), baseline data will be collected for the primary measures, namely interpretation bias and stress reactivity and recovery (see Table 1). After a 30-min acclimation period (during which participants should engage in a pre-approved non-stressful activity of choice), the baseline saliva sample and mood assessment will be collected. Next, the Trier Social Stress Test for Children (TSST-C) [82] will be administered (see below for more details). Directly after the TSST-C [82] the second saliva sample and mood assessment will be collected. Next, participants should complete the first part of the scrambled sentences task (SST, see below for details on this outcome measure) [30]. Subsequently, the third saliva sample and mood assessment will be collected at 10 min post TSST-C. After this, the second part of the SST will be completed. Finally, the fourth, fifth and sixth saliva samples and mood assessments will be collected at 20, 30 and 45-min post TSST-C stressor, respectively. After the assessment of primary outcome variables, children will complete the three questionnaires measuring confounding variables on symptoms of depression and anxiety, childhood trauma and sexual maturation (see Table 1 for the instruments used). When all variables are assessed, HR individuals will commence with the first component (cognitive reappraisal of interpretations component) of the IB intervention (see details on the intervention below). HR participants then conduct the first session of CBM-I training on their smartphones in the lab to assist them with any difficulties that may arise during the first app-based training session. Over the next four weeks, HR individuals will complete components two (CBM-I training) and three (if–then plans) of the IB intervention at their homes or elsewhere (see below for details).

**Table 1:** Overview of screening and outcome measures

Purpose	Construct	Instrument / Response Indices	Session
Inclusion criteria	Parental diagnosis or no diagnosis	DIPS interview	T0
	Child no diagnosis	K-DIPS interview (parent + child report)	T0
Outcome measures	Interpretation bias	SST (computer task)	T1, T2
	Physiological stress reactivity	Cortisol delta (via cortisol swabs at pre, 10 min post, 20 min post and 30 min post stressor)	T1, T2
	Physiological stress recovery	Cortisol levels at 45 min post stressor	T1, T2
	Subjective stress	SAM (self-reported change in current mood measured at pre, 10 min post, 20 min post, 30 min post and 45 min post stressor)	T1, T2
Confounding variables	Pubertal status	Tanner self-report and saliva sample	T1
	Depressive symptoms	R-CADS (self-report)	T1
	Anxiety symptoms	R-CADS (self-report)	T1
	Childhood trauma	CTQ (self-report)	T1

*CTQ* Childhood Trauma Questionnaire [77], *DIPS* Diagnostic Interview for Mental Disorders [78], *K-DIPS* Diagnostic Interview for Mental Disorders in Children and Adolescents [79], *R-CADS* Children’s Anxiety and Depression Scale [80], *SST* Scrambled Sentences Task [30], *SAM* Self-Assessment Manikin Scale [81]

Finally, after the four-week training period, HR individuals will be scheduled for a third session (T2). This post- intervention outcome assessment session will be structurally identical to the pre-intervention assessment at T1, with an adapted version of the TSST-C being administered as well as the version of the SST which they did not complete at T1.

### The interpretation bias intervention

IB will be modified using a four-week primarily app-based IB intervention, which consists of a cognitive reappraisal of interpretations component, a CBM-I component and if-then plans. Participants will be blinded as to which condition (positive vs. placebo) they are allocated to, outcome assessors will not.

#### Cognitive reappraisal of interpretations component

The cognitive reappraisal training will be conducted in the lab and serves as an introduction to the topic of interpretation biases and to the exercises comprised in the CBM-I training. The method used in this study was adopted from a previous study [83] and translated into German. In the cognitive reappraisal of interpretations training participants are explained what negative interpretations are, how they can be modified and how this can have positive effects on mood. This will be executed in a 15-min face-to-face session using cognitive-behavioural therapy techniques. Through a series of practice exercises, participants will be presented with ambiguous social scenarios and taught how to generate fewer negative interpretations and more positive interpretations to change their emotional response. These practice exercises will allow experimenters to determine whether participants have grasped the concept of the task before continuing with the independent CBM-I training. The positive reappraisal condition has previously shown to lead to more neutral interpretations, less negative interpretations, and more positive mood from pre-to post-intervention, when compared to the control condition [83].

The control condition matches the length and difficulty of cognitive reappraisal task but contains no emotional content. In this condition, participants are taught how to convert degrees Fahrenheit into Celsius. This task is matched in format, length, and difficulty to the positive reappraisal condition [83].

#### CBM-I training component

The first app-based CBM-I training session will take place in the lab, followed by four weeks (approx. five weekly sessions) of training from their homes or elsewhere. This will be an independent practice of what was learnt in the cognitive reappraisal component. The paradigm used for the CBM-I training is an adapted version of the Ambiguous Scenarios Task [84]. Participants are first presented with an ambiguous scenario. On the following screen, they are asked what thoughts they would have in this situation and are given two possible answers depicting a positive and a negative interpretation of the situation. Participants will be reinforced for choosing the positive solution. If participants choose the negative solution, they will be notified that there is a more helpful choice. This feedback will be provided in various formats. In line with previous research [85], a total of 500 scenarios will be delivered over four weeks. To better cater to the young sample, the 500 scenarios will be delivered over 20 sessions with 25 scenarios per session (rather than 10 sessions with 50 scenarios per session as in [85]), after receiving qualitative feedback that it was quite strenuous to complete all 50 scenarios. Furthermore, we chose to present the scenarios in text form, accompanied by matching, colourful images, again, to better cater to the young sample. The 500 ambiguous scenarios are appropriate for children in the target age range (10–14 years of age) and designed to capture depressogenic cognitive styles. If participants fail to complete the required sessions, they will be reminded of the importance of regular training via email. Participants will be notified that they are welcome to take breaks on their own terms, however, they should finish each session before the next one is scheduled. Participant will be provided with a training schedule to keep track of when the next session is scheduled.

##### Positive CBM-I condition

In line with previous research [85], in the positive CBM-I condition, 76% of the scenarios will be resolved positively, 12% will be resolved negatively and 12% will remain unresolved. Participants will receive feedback on the helpfulness of their response on all but the 12% of unresolved trials.

##### Placebo CBM-I condition

In the placebo CBM-I training the same scenarios will be presented as in the positive CBM-I condition. However, in 50% of the trials participants will not receive feedback on the helpfulness of their answer choices (no training). These items will be structurally identical to the 12% of unresolved trials in the positive CBM-I training. For the remaining 50% of items, participants will be presented with two answer choices based on factual elements of the scenario (rather than negative and positive interpretations of the scenario). These factual items will be followed by feedback on the accuracy of participants’ answer solutions. Participants in the placebo CBM-I training will be given access to the positive CBM-I training after study completion.

#### If–then plans component

The third component of the IB intervention are if–then plans (e.g., “if my friend is late to meet me at the park, then I won’t take it personally”). Participants will be provided with a colourful sheet where they can fill in each plan and a CoCo merchandise fridge magnet to hang up the sheet in their homes. If–then planning, also known as implementation intentions, reinforce the relationship between the expected situation and behaviour, so that when relevant cues appear, goal-relevant behaviour automatically occurs [86]. Thus, if–then plans reduce the gap between goal and action. A meta-analysis found if–then planning to be a successful tool for behaviour change in terms of achievements, relationships and health, with effect sizes (ES) ranging from medium to large [87]. Participants in the active and placebo conditions will receive differing instructions on the if–then plans. Participants in the active condition will be asked to complete weekly IB specific if–then plans, whereas participants in the control condition will be asked to complete non-interpretation bias specific if–then plans. Participants should make a commitment to take an action in a certain situation. An example if–then plan presented to participants in the active condition is, “if my friend doesn’t respond to my text message immediately, then I will interpret this as them likely being busy with other things.” An example if–then plan presented to participants in the placebo condition is, “if I’m in a rush on the way to school in the morning, then I will still wait for the traffic light to turn green.” The demonstrative if–then plan in the instructions for the placebo condition should be as neutral as possible, nevertheless, it is still possible that participants in the placebo condition create positive if–then plans, which could in turn influence certain outcomes. It is, however, less likely that they will create interpretation specific if–then plans, meaning the primary outcome measure of this study should not be affected.

### Measures

See Table 1 for screening and outcome measures as well as confounding variables.

#### Screening measures

The Diagnostic Interview for Mental Disorders (DIPS) [78] will be used to determine whether the parent of children in the HR sample meets inclusion criteria for a current or previous depressive episode (according to DSM-5 criteria) and whether both parents (if applicable) have no history of a disorder that would lead to exclusion from the study (bipolar disorder, psychosis or substance abuse). The DIPS [78] will also be used to establish the psychiatric diagnosis-free status of parents of children in the low-risk sample. The DIPS is a clinician-administered semi-structured interview used to identify both current and past psychiatric diagnoses in adults.

The Diagnostic Interview for Mental Disorders in Children and Adolescents (K-DIPS) [79] will be used to determine whether children have current or past psychiatric diagnosis according to DSM-5 criteria which would lead to exclusion of the study. The K-DIPS [79] is a clinician-administered semi-structured interview used to identify both current and past psychiatric diagnoses in children aged 6–18. Both the DIPS [78] and K-DIPS [79] will be conducted by team members trained in the use of the manual.

#### Outcome measures

The primary outcomes reflecting our hypotheses are IB, stress reactivity and stress recovery. Furthermore, as a secondary outcome measure, we will use Ecological Momentary Assessments (EMA) to sample participants’ current stress perception and stress coping mechanisms two times a week in a naturalist setting.

##### Interpretation bias

The first primary outcome measure is interpretation bias. This will be measured via a computerised version of the SST [30] adapted to encompass depressogenic stimuli. Two randomised versions (version A and version B) of the SST [30] will be administered pre (T1) and post (T2) IB intervention, with approximately four weeks between measurement timepoints. In the SST [30] participants are presented with 36 scrambled sentences containing six words each. They should use five of these words to build a sentence. Each word series contains more than one possible sentence formulation. Thirty of the scrambled sentences are emotional sentences (e.g., “total I winner a loser am”), with one positive and one negative sentence solution. The remaining six scrambled sentences are neutral sentences (e.g., “like watching funny I exciting movies”), with neutral solutions. IB represents the proportion of emotional sentences which were resolved negatively.

The scrambled sentences of version A of the SST were adopted from a previous study conducted by researchers involved in the present study [41]. The emotional sentences in version A are based on the original stimulus set developed by [88], were then translated into German [89], adapted and extended by researchers in the present research group [41]. Version B of the SST was created for the present study. Approximately 93% of the sentences in version B were adapted from version A of the SST, mainly by changing single elements such as the positive or negative valanced words. The remaining 7% of the sentences were directly adopted from a further study [90].

To avoid deliberate sentence building strategies, participants should complete a simultaneous cognitive load task [30]. Participants are presented with a four-digit number at the beginning of each of three blocks for 5,000 ms, which they should memorise and recall at the end of the block. The split-half reliability (odd vs. even trials) of version A of the SST was found to be acceptable (*r* = 0.53; *p* < 0.001) [41]. Version B was created to closely resemble version A.

##### Stress reactivity and recovery

Stress will be induced using the TSST-C [82]. The adult version (TSST) is the gold standard for inducing stress and reliably increases cortisol levels by two to four times [46]. The TSST-C [82] has been validated for children aged 8–14 years. In this task, participants are asked to finish a story and to solve a mental arithmetic problem (sequential subtraction) in front of an audience of two. Both tasks should last a duration of five minutes [82]. We were able to detect a significant change in cortisol from pre to 30- minutes post TSST-C in a small pilot study (*N* = 6), using the Wilcoxon Rank- Sum test (*Z* = 21, *p* = 0.028). See below for details on data processing and computation of cortisol response indicators.

Subjective stress response will also be assessed through participants’ change in current mood, using the 9-point affective valence scale of the Self-Assessment Manikin (SAM, [81]). SAM [81] is a pictorial scale, depicting a range of emotions from a smiling, happy figure to a frowning, unhappy figure. SAM has been found to be highly correlated with ratings obtained using more lengthy and verbal measures of subjects’ emotional response to an event and thus is a much more rapid yet effective instrument [91]. SAM will be administered immediately after each cortisol swab is taken, meaning the measurement timepoints [81] are the same as those for the saliva swabs; Pre-TSST-C [82], immediately post, 10 min post, 20 min post, 30 and 45 min post-TSST-C [82].

##### Ecological momentary assessment of stress

During the four-week app-based IB intervention, ecological momentary assessment (EMA) will be used to sample participants’ 1) stress on a given day, 2) how well they could cope with this stress and 3) if they were able to use the strategies learnt via the IB intervention to cope with stress. A questionnaire containing the above-mentioned questions will be sent to participants smartphones twice a week over a four-week period. EMA has several advantages over traditional pre- and post-measurement methods. One such advantage is the ecological validity it offers, as assessment takes place in real life situations rather than in the laboratory [92, 93]. Furthermore, EMA offers assessment of within-person variability across time, while individual characteristics are kept constant (e.g., sex, race/ ethnicity, and genotype) [94].

### Confounding variables

#### Pubertal status

Pubertal status has been found to be related to HPA axis activity, with cortisol levels increasing at later pubertal developmental stages [95]. Tanner’s Sexual Maturation Scale (SMS) [96, 97] will be used to assess participants pubertal status. According to a meta-analysis by Campisi et al. [98], a self-report of the Tanner Stages is suitable for reliably finding out whether the subjects are in an early or late pubertal stage. In the Tanner children are presented with a series of pictures depicting different pubertal developmental stages. Participants should select the image which best matches their stage of development. Differing versions will be given to boys and girls. An extremely high inter-rater reliability (100% for breast and 98% for pubic hair) was found in the comparison between a self-assessment by the subjects compared to a physical examination by trained nurses or paediatricians [99]. The self-reported developmental stage will be compared and validated with participants’ salivary hormone levels (estradiol, progesterone and testosterone), since these correlate positively with the Tanner Stages [100].

#### Symptoms of depression

The low mood subscale of the German translation [101] of the Revised Children’s Anxiety and Depression Scale (R-CADS) [80] will be used to assess whether children display depressive symptomology, since both IB and cortisol reactivity may be influenced by symptoms of depression. The R-CADS [80] is a 47-item self-report questionnaire with response options depicted on a 4-point Likert scale. The R-CADS [80] is made up of six subscales. The German version of the depression subscale was found to have good internal consistency (Cronbach’s α = 0.87) [101]. And was strongly associated with the Depression Inventory for Children and Adolescents (DIKJ), *r* = 0.78, showing good convergent validity [101].

#### Symptoms of anxiety

The sum of the five anxiety subscales of the German translation [101] of the Revised Children’s Anxiety and Depression Stress Scale (R-CADS) [80] will be used to assess whether children display symptoms of anxiety, since both IB and cortisol reactivity may be influenced by symptoms of anxiety. The anxiety subscales of the German version of the R-CADS [80] were found to have good internal consistency (Cronbach’s α = 0.94) [101]. The relationship between the German version of the R-CADS [80] anxiety subscales and the Anxiety Questionnaire for Pupils (AFS) [102] and the total score of the Spence Children's Anxiety Scale (SCAS) [103] was found to be strong, *r* = 0.81 and *r* = 0.94 respectively, showing good convergent validity [101].

#### Childhood trauma

The German version [104] of the Childhood Trauma Questionnaire (CTQ) [77] will be used to assess whether participants have experienced trauma, since childhood trauma has previously been found to be associated with distinct salivary cortisol patterns [105]. The CTQ [77] is a 28-item self-report questionnaire assessing retrospective episodes of physical, sexual, and emotional abuse. The psychometric properties of the CTQ [77] have been assessed in both clinical [106, 107] and community samples and were found to be good [108].

### Statistical analyses

#### Cortisol data analyses

Stress reactivity and recovery will be measured via cortisol obtained from saliva swabs pre (T1) and post (T2) the IB intervention. During each assessment session (T1 and T2), cortisol will be sampled at six timepoints; Pre-TSST-C [82], immediately post, 10 min post, 20 min post, 30 min post and 45 min post-TSST-C [82]. The baseline cortisol measurement (pre TSST-C [82]) will be taken after a 30-min acclimation period. This is necessary so that the initial cortisol measurement is reflective of basal cortisol, rather than the stress response associated with part-taking in an experiment in an unfamiliar laboratory setting [18]. Testing will take place between 2 and 7 pm to maximize feasibility by having a reasonable time window and minimise the time of the day effect which is evident in cortisol studies [109].

#### Cortisol data preparation

First, we will check the distributional characteristics of the baseline cortisol data (i.e., cortisol levels pre-TSST-C at session 1). It is expected that some participants will have high baseline cortisol concentrations and that these participants will be unable to elicit a cortisol response to the laboratory stressor (suppression of HPA axis response due to negative feedback [110]). Without knowing the distribution of cortisol values, it is difficult to determine a numerical cut-off. Therefore, we plan to exclude participants whose baseline cortisol levels are significantly higher from other cases in the distribution. We will transform the remaining cortisol data into standardized values (z-scores) and check for outliers by inspecting the respective histograms for each cortisol variable. We will consider z-scores above + 3.29 or below -3.29 as outliers. Once outliers are removed, we will determine the normality of all cortisol variables using the Shapiro–Wilk-Test. If more than one cortisol variable fails the test for normality, all cortisol values will be log transformed before further analyses are conducted.

#### Computation of response indices

We will first compute response indices for both TSST-Cs (pre and post intervention) according to the following procedures: Stress reactivity will be calculated via delta cortisol (i.e., the maximum cortisol increase) by subtracting peak cortisol values (measured either at + 1, + 10, + 20 or + 30 min post-TSST-C) from baseline cortisol values (cortisol measured at -1 min relative to the TSST-C). This has been shown to be a more direct measure of stress reactivity [48] compared to other techniques such as the commonly used AUC method [50]. Stress recovery will be analysed via the salivary cortisol swab taken at 45 min post stressor, correcting for baseline cortisol. The somewhat more intuitive measure of recovery (delta between peak and 45 min post stress) would likely be highly correlated with and driven by cortisol reactivity [111].

#### Hypothesis testing

To test the hypotheses that HR children have more negative IB (H1) and altered cortisol reactivity and recovery (H2, H3) when compared with LR children at T1, t-tests will be performed using IB, cortisol reactivity and recovery as dependant variables (DVs) and group membership (HR, LR) as the independent variable (IV). No specific predictions about the direction of effects for participants’ cortisol reactivity (H2) are made given the mixed literature [18]. For cortisol recovery (H3), it is predicted that HR participants’ cortisol levels will take longer to return to baseline after experiencing the laboratory stressor [47, 111]. Furthermore, to account for the possible confounding variables assessed in this study, a regression model will be run for the effect of group on IB, including age, depression and anxiety symptoms, childhood trauma, sex and pubertal status as covariates. The association between baseline IB and stress response will be looked at within each group (HR and LR) as well as in the entire sample using bivariate correlation. Bayes Factors will be calculated to warrant a rejection of the null hypothesis.

To determine whether the IB training was indeed more effective than the placebo training (H4) in HR individuals, a manipulation check will be conducted using ANOVA. In line with a previous study, participants who complete less than 80% of the training will be excluded from analyses [85].

To test the hypothesis, that the active (versus placebo) IB condition exerts effects on HR individuals’ stress reactivity and recovery (H5 and H6), an ANOVA will be conducted with the condition as the IV (active vs. placebo IB intervention) and cortisol reactivity and recovery as the DVs. Exploratory analyses will be conducted to determine the relationship between change in IB and change in stress response in HR individuals. All relevant confounding variables assessed in this study will be accounted for (age, symptoms of depression and anxiety, childhood trauma, sex, pubertal status, baseline cortisol response). Additionally, Bayes Factors will be calculated to determine whether the null hypothesis can be rejected.

### Sample size

ES for CBM-I to alter IB are moderate to large in youth (*g* = 0.52–0.70) [112]. The ES of CBM-I on cortisol stress response in high-risk youth is difficult to estimate due to inconsistent findings and the lack of research on this specific topic in general. One meta-analysis of studies with youth found a small effect of CBM-I on self-reported anxiety following a stressor (*g* = 0.34) [112]. However, this effect is likely an underestimate as the studies often involved just 1 or 2 training sessions and samples were largely healthy. Furthermore, CBM-I research in an adult sample found that while positive interpretation training did improve recovery from stress as indexed by physiological changes (heart rate, *f* = 0.58), self-reported stress reactivity remained unchanged between conditions [66].

Calculations using GPower ® software [113] yield a minimum sample size of *N* = 70 participants for the comparison of participants’ stress response from pre- to post-intervention between the two intervention groups, using *g* = 0.34 [112], an α = 0.05 and a ß = 0.80 for two conditions and two measurement time points. A dropout rate of 20% should be accounted for in intervention studies. Overall, this results in a total sample of 88 HR participants at T1. Considering the novelty of the research question and the mixed literature, we decided to be particularly cautious and aim for a sample size of 100 HR participants (*n* = 50 per condition).

## Discussion

Although the lifetime prevalence for children of depressed parents developing depression themselves is up to three times higher than in the general population, there is great potential in improving the support which is available to this vulnerable group. Understanding the underlying mechanisms and how these interact could help reinforce the movement away from interventions comprising general psychotherapeutic tools and towards more targeted prevention. This protocol describes an RCT designed to examine how two proposed mechanisms distinguishing children of depressed parents from low-risk children interact. This will be done by targeting one of these mechanisms, namely IB, through a four-week IB intervention and then determining whether this has a positive effect on individuals’ psychobiological stress response. The proposed RCT will therefore be the first to experimentally test the association between cognitive and physiological vulnerability factors in HR youth.

One-hundred HR individuals will be randomly allocated to receive either an i) IB intervention or a structurally and temporally similar ii) placebo intervention. We hypothesise that in comparison to the placebo intervention, the IB intervention will be effective in significantly improving participants’ IB, which will in turn influence their psychobiological stress reactivity and recovery.

We expect children to benefit greatly from the IB intervention. Through more positive interpretation in ambiguous situations, positive effects on their social life, self-efficiency and physical stress experience are to be anticipated. If this is indeed the case, this approach could be used not only as an add-on to Cognitive Behavioural Therapy for children with depression but also as a preventive measure before the onset of the disease [114–116].

The ability to interpret ones environment more positively could lead to an improved stress response and ultimately may heighten the threshold to the onset of the disease itself. Furthermore, considering IB interventions can be delivered digitally (e.g. on handheld devices) they represent a potentially useful resource for universal or selected prevention [67, 117].

There is also the possibility that the intervention modifies IB but has no effect on participants’ physiological stress reactivity. This would indicate that IB does not have a strong casual role in an altered stress responses. On a clinical level, this would indicate that IB interventions may be limited in their efficacy and that alternative approaches, such as a more explicit training during a stressful event may be more beneficial. Regardless of the findings, the proposed RCT will have important theoretical implications for existing models of the familial transmission of depression, which act as a theoretical foundation for many intervention studies.

### Risks and side effects

Existing studies using interpretation bias interventions, provide no evidence of any associated risks or complications. Despite this low-risk, spontaneously reported side- effects of the intervention will be documented and discussed within the team. Moreover, in case of any mental health issues detected during the screening of children, the parents will be informed and advised to seek help for their children. The extensive diagnostic screening should ensure that children with mental illnesses and / or other treatment needs are not included in the study. Children and their parents have the right to withdraw from participation at any point without disadvantage.

## Data Availability

Not applicable.

## Abbreviations

AB: Attentional Bias
AFS: Anxiety Questionnaire for Pupils
AUC: Area Under the Curve
CBM-I: Cognitive Bias Modification of Interpretations
CTQ: Childhood Trauma Questionnaire
DIPS: Diagnostic Interview for Mental Disorders
DSM-5: Diagnostic and Statistical Manual of Mental Disorders
EMA: Ecological Momentary Assessments
ES: Effect Size
HPA: Hypothalamic–pituitary–adrenal
HR: High Risk (children of parents with depression)
IB: Interpretation Bias
K-DIPS: Diagnostic Interview for Mental Disorders in Children and Adolescents
LMU: Ludwig-Maximilians-University
LR: Low Risk (children of parents with no history of mental health issues)
MB: Memory Bias
RCT: Randomised Controlled Trial
R-CADS: Revised Children’s Anxiety and Depression Scale
SCAS: Spence Children's Anxiety Scale
SMS: Tanner’s Sexual Maturation Scale
SPIRIT: Standard Protocol Items: Recommendations for Interventional Trials
SST: Scrambled Sentences Task
TSST: Trier Social Stress Test
TSST-C: Trier Social Stress Test for Children
